# Use of high‐flow nasal cannula oxygen therapy for patients with terminal cancer at the end of life

**DOI:** 10.1002/cam4.6060

**Published:** 2023-05-10

**Authors:** Jung Sun Kim, Jeongmi Shin, Nam Hee Kim, Sun Young Lee, Shin Hye Yoo, Bhumsuk Keam, Dae Seog Heo

**Affiliations:** ^1^ Department of Internal Medicine Chungnam National University Sejong Hospital Sejong Korea; ^2^ Public Healthcare Center Seoul National University Hospital Seoul Korea; ^3^ Center for Palliative Care and Clinical Ethics Seoul National University Hospital Seoul Korea; ^4^ Department of Medicine Seoul National University College of Medicine Seoul Korea; ^5^ Department of Internal Medicine Seoul National University Hospital Seoul Korea; ^6^ Patient‐Centered Clinical Research Coordinating Center National Evidence‐based Healthcare Collaborating Agency Seoul Korea

**Keywords:** end‐of‐life, high‐flow nasal cannula, nasal cannula, neoplasm, oxygen inhalation therapy, retrospective study

## Abstract

**Background:**

Few studies have focused on high‐flow nasal cannula (HFNC) usage in the last few weeks of life. The aim of this study was to identify the status of HFNC use in patients with cancer at the end of life and the relevant clinical factors.

**Methods:**

We performed a retrospective cohort study in a tertiary hospital in the Republic of Korea. Among patients with cancer who died between 2018 and 2020, those who initiated HFNC within 14 days before death were included. Patients were categorized based on the time from HFNC initiation to death as imminent (<4 days) and non‐imminent (≥4 days).

**Results:**

Among the 2191 deceased patients with terminal cancer, 329 (15.0%) were analyzed. The median age of the patients was 66 years, and 62.9% were male. The leading cause of respiratory failure was pneumonia (70.2%), followed by pleural effusion (30.7%) and aggravation of lung neoplasms (18.8%). Most patients were conscious (79.3%) and had resting dyspnea (76.3%) at HFNC initiation. Patients received HFNC therapy for a mean of 3.4 days in the last 2 weeks of life, and 62.6% initiated it within 4 days before death. Furthermore, female sex, no palliative care consultation, no advance statements in person on life‐sustaining treatment, and no resting dyspnea were independently associated with the imminent use of HFNC.

**Conclusions:**

Many patients with cancer started HFNC therapy at the point of imminent death. However, efforts toward goal‐directed use of HFNC at the end‐of‐life stage are required.

## Abstract 2

This study illustrates the status of high‐flow nasal cannula use in patients with terminal cancer at the end of life. Efforts to communicate patients' stated goals of care may help enhance goal‐concordant use of high‐flow nasal cannula at the end of life and reduce initiating in an imminently dying state.

## 1 INTRODUCTION

Dyspnea is a prevalent symptom that 10%–70% of patients with cancer experience near death.^1^, ^2^ Despite the advances in unraveling the pathophysiology and diagnostic workup of dyspnea, those in treatment are unparalleled.^3^ Thus, dyspnea still bothers patients with advanced cancer and remains a challenge for physicians.^4^ To date, pharmacologic agents, such as opioids, and non‐pharmacological approaches, such as oxygen therapy, are widely used to manage dyspnea.^3^ In patients with advanced cancer, an initial approach with non‐pharmacologic methods and treatment of underlying causes is recommended.^5^ However, oxygen therapy is narrowly recommended for patients with hypoxemic dyspnea.^5^

Conventional oxygen therapy uses a nasal cannula or facial mask to deliver low oxygen flow. Additionally, non‐invasive ventilation improved gas exchange, but tolerance was poor due to synchronizing difficulty, claustrophobia, and distinctive mask‐related side effects. Therefore, a high‐flow nasal cannula (HFNC) was recently introduced in clinical practice. It supplies a high flow of heated and humidified oxygen via an interface with a silicon nasal cannula without occlusion, enabling patients to talk or eat while on oxygen.^6^ Notably, non‐invasive ventilation is superior to conventional oxygen in reducing dyspnea and opioid doses in patients with terminal cancer who only received palliative care.^7^ Moreover, HFNC is superior to conventional oxygen in alleviating dyspnea and well tolerated in patients with cancer.^8^, ^9^, ^10^

Meanwhile, the role of oxygen and its optimal delivery methods at the end of life has yet to be established.^11^ Even though HFNC is a tolerable option, it is barely suggested in a time‐limited manner when dyspnea is unrelieved by conventional oxygen therapy.^5^ To further develop a consensus on HFNC application at the end of life, understanding the current status of HFNC use is essential. Therefore, we aimed to investigate the status of HFNC use in patients with terminal cancer at the end of life and the relevant clinical factors.

## 2 MATERIALS AND METHODS

### 2.1 Study design

**FIGURE 1:**
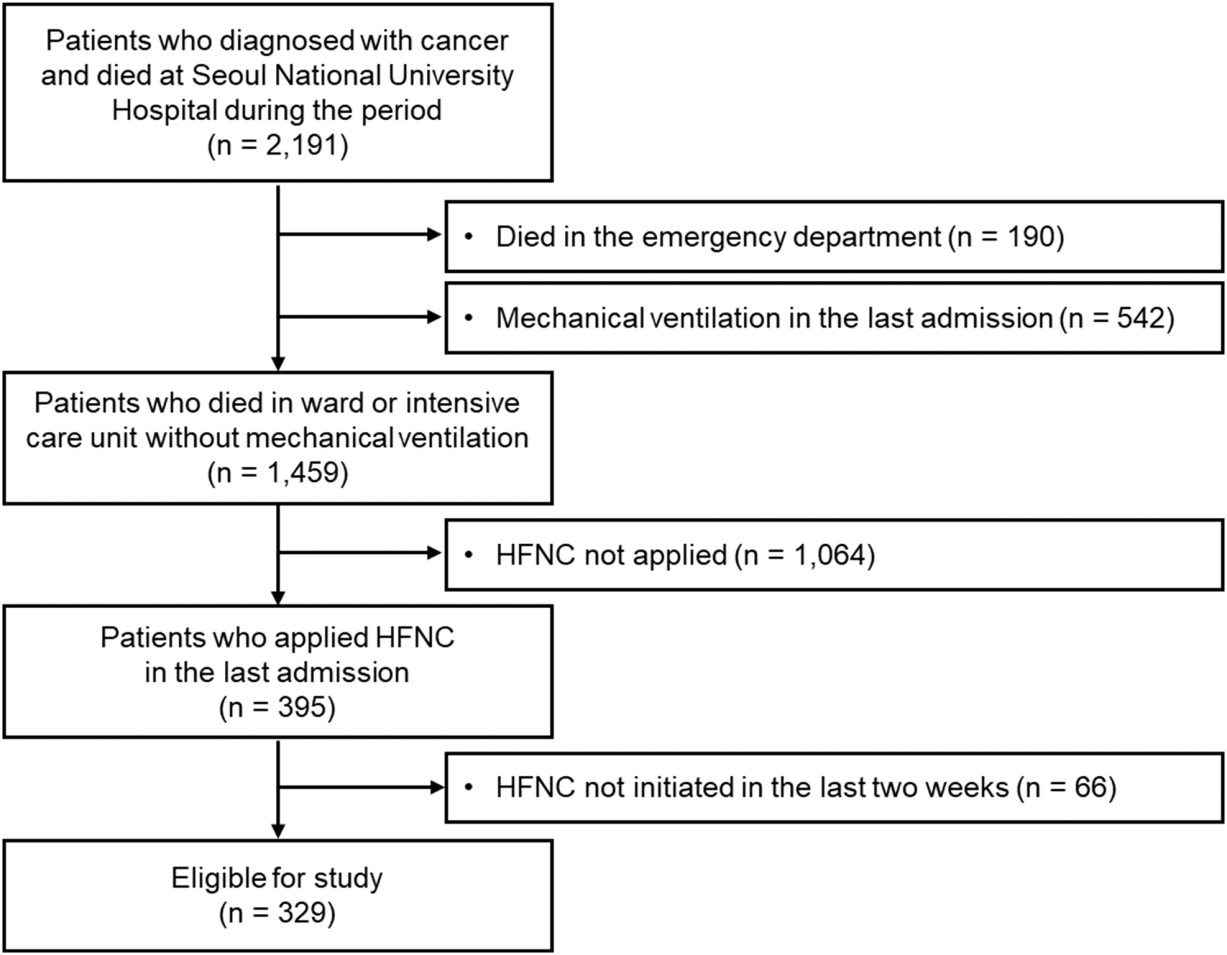
Patient enrollment flow. HFNC, high‐flow nasal cannula; *n*, number.

We conducted a single‐center retrospective study of patients with cancer who died at Seoul National University Hospital (SNUH) between January 2018 and December 2020. SNUH is a tertiary university hospital with 1751 beds and 1800 doctors in the Republic of Korea that does not operate in an inpatient hospice‐palliative care ward. We reviewed data based on the last admissions before death to evaluate HFNC usage in the do‐not‐intubate setting at the end of life. Next, we excluded patients who died in the emergency department and those who underwent mechanical ventilation during admission. Patients who did not receive HFNC were excluded. Additionally, patients who did not initiate HFNC therapy 14 days before death were excluded as the “last 14 days” was one of the time criteria for end‐of‐life cancer care's intensity (Figure 1).^12^

Additionally, the study period was prior to the emergence of the Omicron variant of coronavirus disease 2019 (COVID‐19) when infected patients received treatments at hospitals dedicated to COVID‐19 in Korea. Hence, we supposed that the pandemic would not likely affect patients' care and access to HFNC in SNUH.

### 2.2 Data collection and measurements

Demographic and clinical characteristics at baseline, status during HFNC application, and data regarding HFNC use patterns were obtained from electronic medical records. Symptoms and image findings were based on medical records mainly written by internists and formal readings by radiologists, respectively. We collected the data for the department where patients were initially admitted and classified them as “medical” for any subdivision of internal medicine and “non‐medical” for other departments. In addition, we classified patients as recipients of palliative care consultation if they had medical records indicating that they were requested to the palliative care team of SNUH. The team comprises medical oncologists, palliative care nurses, and medical social workers with sufficient clinical experience in palliative care. The consultation was conducted when the primary attending physicians determined it was necessary for the patients' disease course and made a request. During the consultation, the team comprehensively assessed palliative care needs through interviews and underwent discussion for goals of care and advance care planning at the end of life. We evaluated the Charlson comorbidity index^13^ after excluding malignancy‐related conditions such as solid tumors, leukemia, and lymphoma (i.e., non‐cancer CCI). Given that the last 3 days before death is the most common definition of impending death in patients with cancer, we used the time from HFNC initiation to death to categorize into the imminent (<4 days) and non‐imminent (≥4 days) groups.^14^, ^15^ To evaluate short‐term change in pharmacologic measures for dyspnea, we calculated opioid doses within 48 h before and after HFNC initiation using morphine equivalent daily dose (MEDD).^16^

The “Act on Hospice and Palliative Care and Decisions on Life‐Sustaining Treatment (LST) for Patients at the End of Life” was implemented in February 2018 in the Republic of Korea.^17^ It enables patients to make advance statements in person that they do not require LST through advance directives (form number 6) or physician orders for LST (form number 1). Therefore, we considered them as having “advance statements by patients” if there were either form number 1 or 6. Furthermore, at an imminently dying state, specific preferences should also be decided and documented (hereafter, “LST documentation”) for the following treatments: cardiopulmonary resuscitation, mechanical ventilation, hemodialysis, anti‐cancer treatment, transfusion, inotropic agents, and extracorporeal membrane oxygenation. However, the LST document does not explicitly encompass HFNC. Besides, if the patient has no advance statements or cannot express the intention of LST, first‐degree family members should decide on behalf of the patient. In this study, we reviewed the presence of advance statements, LST documentation, and documentation dates.

### 2.3 Statistical analysis

Descriptive data were used to summarize the demographic and clinical characteristics. Pearson's chi‐square or Fisher's exact tests and Kruskal–Wallis analysis of variance were used for categorical and numeric variables to compare groups, respectively. We performed a univariable analysis, and statistically significant variables were included in the multivariable logistic regression analysis with backward selection to identify relevant factors in the imminent use of HFNC. All statistical analyses were two‐sided, and the significance level was set at *p* < 0.05. All analyses were conducted using STATA version 16.0 (StataCorp LP).

## 3 RESULTS

**FIGURE 2:**
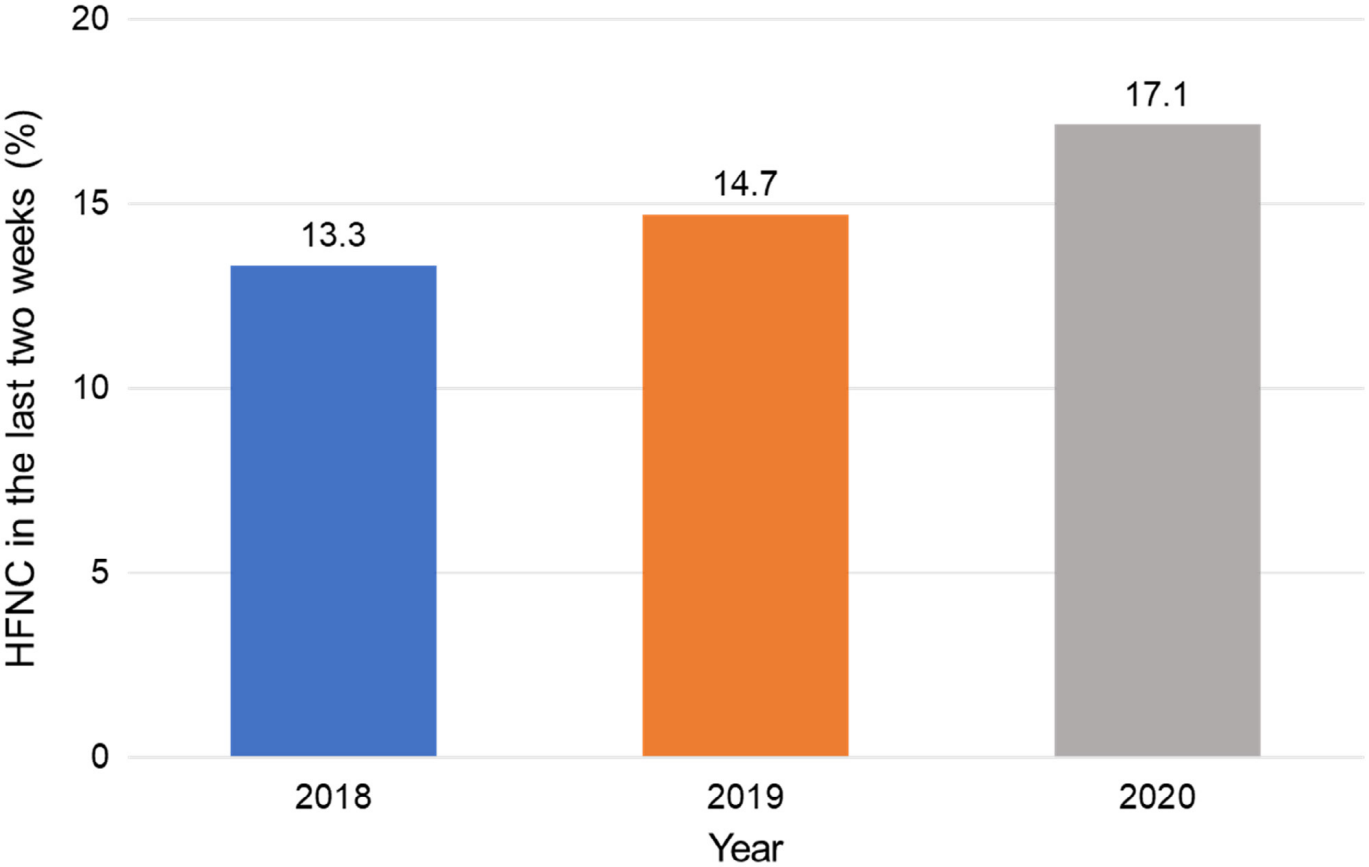
Trends of high‐flow nasal cannula application in the last 2 weeks of life. HFNC, high‐flow nasal cannula.

Among the 2191 patients with cancer who died during the study, 329 initiated HFNC treatment 14 days before death and were included in the final evaluation. The percentage of patients who used HFNC during the last 14 days steadily increased from 13.3% to 17.1% annually (Figure 2). Overall, 62.6% (206/329) and 37.4% (123/329) were in the imminent and non‐imminent groups, respectively.

### 3.1 Patient characteristics

Table 1 shows the baseline characteristics of the imminent and non‐imminent groups. Although evenly distributed, the imminent group was younger (median, 65 vs. 68 years, *p* = 0.031), with a higher percentage of females (41.8 vs. 29.3%, *p* = 0.023) than the non‐imminent group. Moreover, the imminent group stayed in the hospital for a significantly shorter duration (9 vs. 13 days, *p* < 0.001), with a lower proportion of lung cancer (22.8 vs. 33.3%, *p* = 0.037) than the non‐imminent group. Regarding advance care planning, a significantly lower proportion of patients in the imminent group received palliative consultation (44.7 vs. 61.0%, *p* = 0.004) and provided advance statements in person (45.6 vs. 63.4%, *p* = 0.002) than the non‐imminent group.

**TABLE 1:** Baseline characteristics of patients.

	Total (*n* = 329)	Imminent use (<4 days) (*n* = 206)	Non‐imminent use (≥ 4 days) (*n* = 123)	*p*‐value^a^
Age (years), median (IQR)	66 (60–74)	65 (58–73)	68 (63–75)	0.031
Sex, *n* (%)				
Male	207 (62.9)	120 (58.3)	87 (70.7)	0.023
Female	122 (37.1)	86 (41.8)	36 (29.3)	
Insurance^b^, *n* (%)				
National Health Insurance	316 (96.0)	195 (94.7)	121 (98.4)	0.142
Medical aid/None	13 (4.0)	11 (5.3)	2 (1.6)	
Route of admission, *n* (%)				
Outpatient clinics	121 (36.8)	77 (37.4)	44 (35.8)	0.770
Emergency department	208 (63.2)	129 (62.6)	79 (64.2)	
Admission department^c^, *n* (%)				
Medical department	308 (93.6)	192 (93.2)	116 (94.3)	0.817
Non‐medical department	21 (6.4)	14 (6.8)	7 (5.7)	
Diagnosis, *n* (%)				
Lung cancer	88 (26.7)	47 (22.8)	41 (33.3)	0.037
Non‐Lung cancer	241 (73.3)	159 (77.2)	82 (66.7)	
Gastrointestinal cancer	34 (14.1)	18 (11.3)	16 (19.5)	
Hepatobiliary‐pancreas cancer	67 (27.8)	50 (31.5)	17 (20.7)	
Breast and gynecological cancer	29 (12.0)	25 (15.7)	4 (4.9)	
Hematologic disease	71 (29.5)	46 (28.9)	25 (30.5)	
Others^d^	40 (16.6)	20 (12.6)	20 (24.4)	
Disease status, *n* (%)				
Metastatic or recurred	271 (82.4)	169 (82.0)	102 (82.9)	0.838
Non‐metastatic	58 (17.6)	37 (18.0)	21 (17.1)	
Non‐cancer CCI, median (range)	0 (0–9)	0 (0–9)	0 (0–6)	0.652
Length of hospital stay (days), median (IQR)	11 (6–24)	9 (4–24)	13 (9–26)	<0.001
Palliative care consultation, *n* (%)				
Yes	167 (50.8)	92 (44.7)	75 (61.0)	0.004
No	162 (49.2)	114 (55.3)	48 (39.0)	
Advance statement by patients, *n* (%)				
Yes	172 (52.3)	94 (45.6)	78 (63.4)	0.002
No	157 (47.7)	112 (54.4)	45 (36.6)	
LST implementation documented before HFNC, *n* (%)				
Yes	98 (29.8)	68 (33.0)	30 (24.4)	0.098
No (after or none)	231 (70.2)	138 (67.0)	93 (75.6)	
Deathplace, *n* (%)				
General ward	313 (95.1)	194 (94.2)	119 (96.7)	0.428
Intensive care unit	16 (4.9)	12 (5.8)	4 (3.3)	

Abbreviations: CCI, Charlson comorbidity index; HFNC, high‐flow nasal cannula; IQR, interquartile range; LST, life‐sustaining treatment; *n*, number.

^a^ Pearson's chi‐squared test, Fisher's exact test, or Kruskal‐Wallis' analysis was used as appropriate.

^b^ Healthcare system in the Republic of Korea has two components: (1) National health insurance to provide coverage to all citizens, managed comprehensively in the form of social insurance and funded by beneficiaries' contributions; (2) Medical aid to provide support to lower income groups, funded by general revenue. Those who are neither citizens of the Republic of Korea nor have obtained health insurance qualifications through the residence for a certain period of time fall into the “None” category.

^c^ “Medical” department for any subdivision of internal medicine, and “non‐medical” department included obstetrics and gynecology, general surgery, emergency department (short stay units only), orthopedics, and thoracic surgery.

^d^ Head and neck cancer (*n* = 5), genitourinary cancer (*n* = 13), thyroid cancer (*n* = 3), central nervous system cancer (*n* = 3), bone and soft tissue cancer (*n* = 14), metastasis of unknown origin (*n* = 1), thymic carcinoma (*n* = 1).

### 3.2 Clinical status at the time of application of HFNC

Pneumonia (70.2%) was the most common etiology of respiratory failure, followed by pleural effusion and lung cancer or metastasis aggravation. The reasons for applying HFNC were balanced between the two groups, with hypoxia, dyspnea, and tachypnea presented in 90.0%, 71.1%, and 49.9% of the population, respectively.

Patients who were alert (75.7 vs. 85.4%, *p* = 0.037) or had resting dyspnea (71.8 vs. 83.7%, *p* = 0.014) were substantially underrepresented in the imminent group compared with the non‐imminent group. Additionally, patients in the imminent group received more oxygen before HFNC (median, 10 vs. 7 L/min, *p* = 0.002) and had significantly lower percutaneous oxygen saturation (SpO2; 88 vs. 89%, *p* = 0.003). No significant difference in the MEDD was observed between the two groups (Table 2).

**TABLE 2:** Clinical status at the time of application of high‐flow nasal cannula.

	Total (*n* = 329)	Imminent use (<4 days) (*n* = 206)	Non‐imminent use (≥4 days) (*n* = 1d)	*p*‐value^a^
Etiology of respiratory failure^b^, *n* (%)				
Pneumonia	231 (70.2)	137 (66.5)	94 (76.4)	0.057
Lung cancer or metastasis aggravation	62 (18.8)	39 (18.9)	23 (18.7)	0.958
Pulmonary edema	38 (11.6)	29 (14.1)	9 (7.3)	0.075
Pleural effusion	101 (30.7)	66 (32.0)	35 (28.5)	0.495
Others^c^	50 (15.1)	34 (16.5)	16 (13.0)	0.393
Reasons for HFNC application, *n* (%)				
Hypoxia				
Yes	296 (90.0)	189 (91.8)	107 (87.0)	0.165
No	33 (10.0)	17 (8.3)	16 (13.0)	
Dyspnea				
Yes	234 (71.1)	140 (68.0)	94 (76.4)	0.101
No	95 (28.9)	66 (32.0)	29 (23.6)	
Tachypnea				
Yes	164 (49.9)	110 (53.4)	54 (43.9)	0.096
No	165 (50.2)	96 (46.6)	69 (56.1)	
Clinical characteristics before applying HFNC				
Consciousness				
Alert	261 (79.3)	156 (75.7)	105 (85.4)	0.037
Drowsy/stupor/semi‐coma, coma	68 (20.7)	50 (24.3)	18 (14.6)	
Resting dyspnea				
Yes	251 (76.3)	148 (71.8)	103 (83.7)	0.014
No	78 (23.7)	58 (28.2)	20 (16.3)	
Pneumonic infiltration at CXR				
Yes	316 (96.1)	196 (95.2)	120 (97.6)	0.277
No	13 (4.0)	10 (4.9)	3 (2.4)	
Vital signs, median (IQR)				
Body temperature (°C)	37 (36.5–37.5)	37 (36.5–37.6)	36.8 (36.6–37.3)	0.274
Mean blood pressure (mmHg)	86.7 (73.3–99.3)	84.7 (69.7–97.3)	90 (79.3–104)	<0.001
Heart rate (beats/min)	115 (98–130)	115 (100–132)	112 (96–126)	0.165
Respiratory rate (breaths/min)	26 (24–32)	28 (24–32)	26 (22–30)	0.274
SpO2 (%)	88 (85–90)	88 (84–90)	89 (87–91)	0.003
O2 flow (L/min)				
Median (IQR)	10 (6–15)	10 (6–15)	7 (6–15)	0.002
≤6, *n* (%)	127 (38.6)	69 (33.5)	58 (47.2)	0.014
>6, *n* (%)	202 (61.4)	137 (66.5)	65 (52.9)	
MEDD within 48 h before applying HFNC (mg), median (IQR)	29 (0–105)	33 (0–120)	18 (0–78)	0.122
Places when applying HFNC				
General ward	301 (91.5)	186 (90.3)	115 (93.5)	0.415
Non‐general ward^d^	28 (8.5)	20 (9.7)	8 (6.5)	

Abbreviations: CXR, chest x‐ray; HFNC, high‐flow nasal cannula; IQR, interquartile range; MEDD, morphine equivalent daily dose; *n*, number; SpO2, percutaneous oxygen saturation.

^a^ Pearson's chi‐squared test, Fisher's exact test, or Kruskal‐Wallis' analysis was used as appropriate.

^b^ Overlap exists between etiologies.

^c^ Count if accounts for any of the following: pulmonary thromboembolism, pneumothorax, atelectasis, aggravation of general condition.

^d^ Intensive care unit or emergency department.

### 3.3 Factors associated with imminent use of the HFNC

In the univariable logistic regression analyses, younger age (*p* = 0.043), female sex (*p* = 0.024), a diagnosis of other than lung cancer (*p* = 0.038), no palliative care consultation (*p* = 0.004), and no advance statement in person (*p* = 0.002) were associated with the imminent use of HFNC. Additionally, non‐alert mental status (*p* = 0.039) and no resting dyspnea (*p* = 0.015) were associated with its imminent use.

Following the multivariable regression model, female sex (odds ratio [OR], 1.72; 95% confidence interval [CI], 1.05–2.84; *p* = 0.032), no palliative care consultation (OR, 1.97; 95% CI, 1.23–3.16; *p* = 0.005), no advance statement in person (OR, 1.66; 95% CI, 1.03–2.67; *p* = 0.038), and no resting dyspnea (OR, 1.94; 95% CI, 1.08–3.51; *p* = 0.027) were independently associated with imminent use of HFNC (Table 3).

**TABLE 3:** Univariable and multivariable analysis for imminent use^a^ of high‐flow nasal cannula.

	Univariable			Multivariable^d^		
Variables	OR	95% CI	*p*‐value	OR	95% CI	*p*‐value
Baseline characteristics						
Age (continuous)	0.98	0.96, 0.99	0.043			
Sex (female vs. male)	1.73	1.08, 2.79	0.024	1.72	1.05, 2.84	0.032
Admission duration (continuous)	0.99	0.98, 1.00	0.088			
Insurance status (National Health Insurance vs. Medical aid/none)	0.29	0.06, 1.35	0.114			
Places when applying HFNC (general ward vs. non‐general ward^b^)	0.65	0.28, 1.52	0.317			
Routes of admission (outpatient clinics vs. emergency department)	0.93	0.59, 1.48	0.770			
Admission department (medical vs. non‐medical department)	0.83	0.33, 2.11	0.692			
Lung cancer diagnosis (no vs. yes)	1.69	1.03, 2.78	0.038			
Disease status (metastatic/recurred vs. non‐metastatic)	0.94	0.52, 1.70	0.838			
Palliative care consultation (no vs. yes)	1.94	1.23, 3.05	0.004	1.97	1.23, 3.16	0.005
Advance statement by patients (no vs. yes)	2.07	1.31, 3.27	0.002	1.66	1.03, 2.67	0.038
LST implementation documented before HFNC (yes vs. no)	1.53	0.92, 2.53	0.099			
Clinical characteristics before applying HFNC						
Consciousness (non‐alert^c^ vs. alert)	1.87	1.03, 3.38	0.039			
Resting dyspnea (no vs. yes)	2.02	1.15, 3.56	0.015	1.94	1.08, 3.51	0.027
Pneumonic infiltration at CXR (yes vs. no)	0.49	0.13, 1.82	0.286			
MEDD within 48 h (median) (lower vs. upper)	0.72	0.46, 1.12	0.143			
Reasons for HFNC application						
Hypoxia (yes vs. no)	1.66	0.81, 3.43	0.168			
Dyspnea (yes vs. no)	0.65	0.39, 1.09	0.102			
Tachypnea (yes vs. no)	1.46	0.93, 2.29	0.096			

Abbreviations: CI, confidence interval; CXR, chest x‐ray; HFNC, high‐flow nasal cannula; LST, life‐sustaining treatment; MEDD, morphine equivalent daily dose; OR, odds ratio.

^a^ Less than 4 days between initial application of high‐flow nasal cannula and death.

^b^ Intensive care unit or emergency department.

^c^ Includes drowsy, stupor, semi‐comatous, comatous state.

^d^ Variables that had significance of *p*‐values <0.05 in the univariable analysis were included in the multivariable analysis (backward stepwise selection).

### 3.4 Patterns of HFNC usage

Table 4 shows that patients used HFNC for a mean duration of 3.4 days in the last 2 weeks of life. Twenty‐two patients (6.7%) received HFNC more than once, and 88.8% (292/329) were under HFNC at death. Among the 37 patients in the HFNC‐free state at death, 37.8% (14/37) were weaned (i.e., tapered off), and 62.2% (23/37) withdrew. Additionally, the mean duration without HFNC was 2.8 days for patients in the HFNC‐free state, with 5.5 days and 1.1 days for those who weaned and withdrew, respectively. The time of the HFNC application did not impact changes in MEDD.

**TABLE 4:** Patterns of high‐flow nasal cannula usage.

	Total (*n* = 329)	Imminent use (<4 days) (*n* = 206)	Non‐imminent use (≥ 4 days) (*n* = 123)
Duration of HFNC application in the last 2 weeks^a^	3.4 ± 3.4	1.2 ± 0.9	7.0 ± 2.7
Number of HFNC application, *n* (%)			
1	307 (93.3)	201 (97.6)	106 (86.2)
2 or more	22 (6.7)	5 (2.4)	17 (13.8)
HFNC application status at death, *n* (%)			
On‐state	292 (88.8)	187 (90.8)	105 (85.4)
Off‐state, *n* (%)	37 (11.2)	19 (9.2)	18 (14.6)
Interval from cessation of HFNC to death^a^	2.8 ± 3.6	0.7 ± 0.8	4.9 ± 4.1
Weaning (tapered off), *n* (%)	14 (37.8)	4 (21.1)	10 (55.6)
Interval from cessation of HFNC to death^a^	5.5 ± 4.1	0.5 ± 0.6	7.5 ± 3.0
Withdrawal, *n* (%)	23 (62.2)	15 (79.0)	8 (44.4)
Interval from cessation of HFNC to death^a^	1.1 ± 1.8	0.8 ± 0.9	1.8 ± 2.8
Changes in MEDD after HFNC^b^ (mg), median (IQR)	0 (−6, 39)	0 (−15, 40)	0 (0, 35)

Abbreviations: HFNC, high‐flow nasal cannula; IQR, interquartile range; MEDD, morphine equivalent daily dose; *n*, number.

^a^ Values are presented as means ± standard deviation (days).

^b^ Calculated as subtraction of ‘Pre 48‐hour MEDD’ from ‘Post 48‐hour MEDD’.

### 3.5 Temporal relationship between HFNC application and LST documentation

**FIGURE 3:**
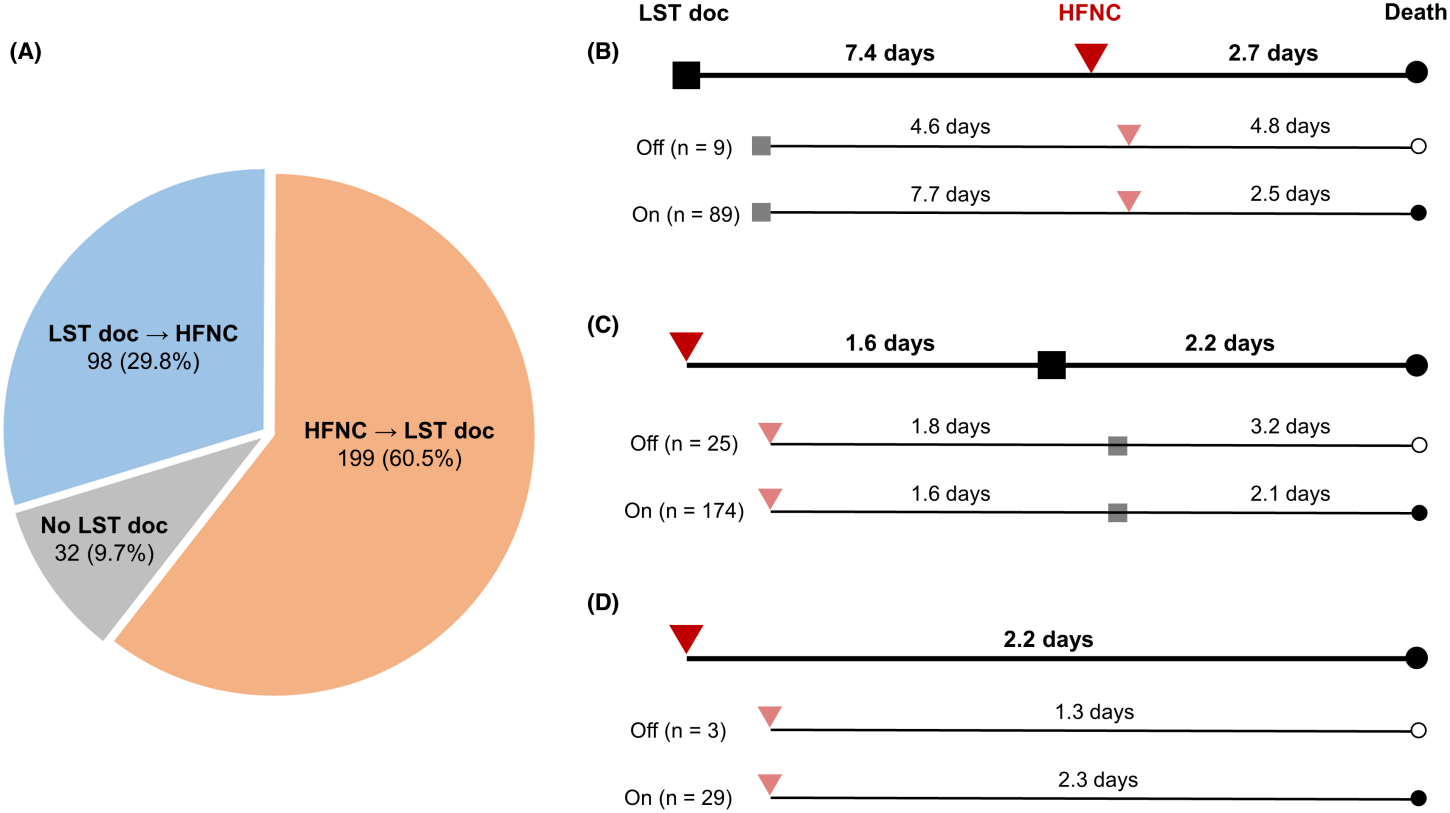
Patterns of high‐flow nasal cannula application and life‐sustaining treatment documentation. (A) Patients' distribution by sequence of life‐sustaining treatment documentation and high‐flow nasal cannula. (B) Application patterns in patients who completed life‐sustaining treatment documentation first, and (C) who applied high‐flow nasal cannula first. (D) Application patterns in patients without life‐sustaining treatment documentation. Mean values are presented in (B–D). HFNC, high‐flow nasal cannula application; LST, life‐sustaining treatment documentation; *n*, number.

We evaluated patients' temporal relationships to delineate how they completed LST documentation and practically applied HFNC. Accordingly, 29.8% (98/329) 60.5% (199/329), and 9.7% (32/329) completed LST documentation before HFNC, applied HFNC and completed the documentation and experienced no complete documentation, respectively (Figure 3A).

For patients who completed LST documentation first, the mean interval from documentation to HFNC application was 7.4 days, and they had additional 2.7 days until death (Figure 3B). Next, patients who started HFNC first had a mean interval of 1.6 days before completing LST documentation and an additional 2.2 days until death (Figure 3C). Lastly, for patients who died without complete LST documentation, the mean interval from HFNC application to death was 2.2 days, which was shorter than that of the two previously mentioned groups (Figure 3D).

## 4 DISCUSSION

Our study focused on using HFNC in patients with terminal cancer during the last 2 weeks of life. Notably, 62.6% of HFNC users started treatment when their death was imminent. Female sex, no palliative care consultation, no advance statement in person, and no resting dyspnea were associated with the imminent use. Additionally, the average duration of HFNC use was 3.4 days, whereas its duration in the non‐imminent use group was approximately a week. Even though the palliative use of HFNC has recently gained attention,^8^, ^10^, ^18^ it remains questionable whether such a brief administration at the time of death is appropriate.

Most patients showed hypoxia or dyspnea and had an acute cause, such as pneumonia, when initiating HFNC. This finding is consistent with previous studies^19^, ^20^, ^21^, ^22^ and supports what other physicians consider as indications of HFNC.^19^ Then, it reflects the features of an acute‐care hospital, even for patients with cancer at the end of life. We also observed that most patients maintained HFNC until death, with only a few opting for withdrawal. However, insufficient evidence shows the benefit of high‐flow oxygen in patients dying from respiratory failure for either substantial survival prolongation or symptom relief, regardless of the underlying reason.^21^, ^23^ In addition, prolonged use of HFNC did not significantly lower opioid usage in this real‐world practice. Therefore, setting a clear goal before applying HFNC is valuable.

Several clinical signs appear in the last days of life in patients with advanced cancer.^14^, ^15^, ^24^ A decrease in oxygen saturation is one of them, which can be taken as a natural dying process,^24^ and mere correction with oxygen at this stage would do more for life‐sustaining than alleviating symptoms. Moreover, when patients reach this stage, evaluating efficacy by a “time‐limited trial^5^” is less valuable as patients tend to become unconscious. In a recent study at another tertiary hospital, HFNC weaning for patients with cancer at the end of life was difficult; only a minority were liberated from it, even with their protocol.^25^ Thus, we assume that the non‐imminent use of HFNC is beneficial in securing time for a time‐limited trial and discussion for goals of care at the end of life.

Accordingly, the presence of palliative care consultation or advance statements by the patients may lead to more beneficial usage of HFNC. Considering prior studies that palliative care consultation helped patients receive less aggressive end‐of‐life care via goals‐of‐care discussions,^26^, ^27^ the non‐imminent group may have benefitted from end‐of‐life discussion and not solely sustain life. In this context, no palliative care consultation could lead to HFNC initiation in the imminent dying state. In addition, no prior documentation on the advance statement in person would make it harder for patients, their families, and physicians a chance to make shared decisions to avoid imminent use or prompt withdrawal of HFNC, currently in the gray zone. These findings imply that advance care planning may help the optimal use of HFNC at the end of life.

Despite the growing interest in the influence of sex differences in clinical studies, the relationship between sex differences and end‐of‐life care for patients with cancer is not well understood. In this study, we observed that female patients were likely to initiate HFNC therapy at imminent death. This finding contradicts a previous study, which showed that male patients receive more aggressive end‐of‐life care than females, such as intensive care units.^28^ In a setting where we excluded those who applied mechanical ventilation, a core part of intensive care units, female patients may seem to be receiving more aggressive care than males, such as the imminent use of HFNC. Therefore, additional studies regarding sex disparities in HFNC use would help clarify this issue.

Our study showed that no resting dyspnea was associated with the imminent use of HFNC. Assumably, physicians would take it as they are not in the process of imminent death and apply HFNC without much concern. Although the level of consciousness may affect how patients complain of their discomfort, the multivariable analysis revealed that no dyspnea at rest was an independent factor associated with the imminent use of HFNC.

An essential aspect of using HFNC to alleviate dyspnea in patients with cancer is whether it adheres to the individual goal, as the American Society of Clinical Oncology guidelines recommended.^5^ Meanwhile, the intensive care unit (ICU) admission rate of cancer patients at the end of life in Korea increased steadily, reaching 30% in the last month reported at a tertiary referral hospital by retrospective data,^29^, ^30^ and 20% in the last 6 months before death by national claim data.^31^ As end‐of‐life care still being aggressive, we believe this is a good starting point to discuss the appropriate use of HFNC at the end of life. When used appropriately, HFNC could ease the patient's symptoms without requiring additional invasive ventilation.^32^, ^33^, ^34^ If not, some may lose the chance of recovery by not receiving HFNC properly, while some merely extend an undesirable life by it. Since HFNC remains a scarce medical resource in hospitals, its unnecessary use may conflict with the issue of distributive justice from an ethical standpoint.^35^

From the physician's perspective, however, various concerns exist in applying HFNC, such as the need to gain time for LST documentation, uncertainty about the reversibility of the patient, or demands for HFNC application by patient families who find it difficult to accept their loved one's death.^35^, ^36^, ^37^, ^38^, ^39^ Hence, we suggest delicately exploring the patient's goals, values, and preferences through serial conversations with stakeholders regarding the HFNC application. Furthermore, time‐limited trials within a few hours can help decide the continuation or withdrawal of HFNC.^5^, ^40^, ^41^ Moreover, compassionate removal should also be considered to allow natural death if HFNC use does not achieve the goal and when it has only a minor practical effect.^42^

As we analyzed a relatively homogeneous population of patients with terminal cancers at the end of life, we expect this study to provide fundamental data for developing guidelines for HFNC use at the end of life. However, there are some limitations in this study. First, it was performed in a single tertiary hospital primarily focused on the acute care of patients; therefore, we should be cautious in generalizing our results. Second, dyspnea and hypoxemia could not be evaluated objectively, so data regarding HFNC usage in specific populations with hypoxemic dyspnea is limited. Lastly, because of the study's retrospective nature, it was difficult to evaluate the patients stated goals of care thoroughly. Therefore, further well‐designed, large prospective studies assessing objective findings and goal‐directed use are warranted to overcome these limitations.

## 5 CONCLUSION

Many patients with cancer who underwent HFNC at the end of life initiated it in an imminently dying state. Additionally, inadequate advance care planning was likely associated with the imminent use of HFNC. Therefore, it is necessary to communicate patients' stated goals of care in advance and to work toward goal‐directed use of HFNC at the end of life.

## AUTHOR CONTRIBUTIONS

**Jung Sun Kim:** Conceptualization (equal); data curation (equal); formal analysis (equal); visualization (equal); writing – original draft (equal); writing – review and editing (equal). **Jeongmi Shin:** Conceptualization (equal); data curation (equal); formal analysis (equal); resources (equal); writing – original draft (equal); writing – review and editing (equal). **Nam Hee Kim:** Data curation (equal); resources (equal); writing – review and editing (equal). **Sun Young Lee:** Conceptualization (equal); writing – review and editing (equal). **Shin Hye Yoo:** Conceptualization (equal); data curation (equal); formal analysis (equal); investigation (equal); methodology (equal); resources (equal); supervision (equal); writing – original draft (equal); writing – review and editing (equal). **Bhumsuk Keam:** Supervision (equal); writing – review and editing (equal). **Dae Seog Heo:** Supervision (equal); writing – review and editing (equal).

## CONFLICT OF INTEREST STATEMENT

The authors declared no potential conflicts of interest with respect to the research, authorship, and/or publication of this article.

## ETHICS APPROVAL STATEMENT

The study protocol was reviewed and approved by the Institutional Review Board of SNUH (No. H‐2203‐120‐1308) and conducted following the principles of the Declaration of Helsinki. Additionally, the SNUH Institutional Review Board waived the requirement for informed consent due to the study's retrospective nature.

## Data Availability

The data that support the findings of this study are available from the corresponding author upon reasonable request.
